# A randomised controlled trial of plasma exchange compared to standard of care in the treatment of severe COVID-19 infection (COVIPLEX)

**DOI:** 10.1038/s41598-024-67028-3

**Published:** 2024-07-23

**Authors:** Nishkantha Arulkumaran, Mari Thomas, Matthew Stubbs, Nithya Prasanna, Maryam Subhan, Deepak Singh, Gareth Ambler, Alessia Waller, Mervyn Singer, David Brealey, Marie Scully

**Affiliations:** 1 Intensive Care Unit, University College London Hospitals NHS Foundation Trust, London, UK; 2 Bloomsbury Institute of Intensive Care Medicine, University College London, London, UK; 3 Department of Haematology, University College London Hospitals NHS Foundation Trust and Haematology Programme-NIHR UCLH/UC BRC London, 235 Euston Road, London, NW12PG UK; 4 Department of Haematology, University College London Hospitals NHS Foundation Trust, London, UK; 5 Special Coagulation, UCLH-HSL, London, UK; 6 Department of Statistical Science, University College London, London, UK

**Keywords:** Coagulation system, Infection

## Abstract

COVID-19 disease is associated with a hyperinflammatory, pro-thrombotic state and a high mortality. Our primary objective was to assess the change in inflammatory and thrombotic markers associated with PEX, and secondary objectives were to assess the effects of PEX on progression of respiratory failure and incidence of acute thrombotic events. We conducted a prospective, phase II, non-blinded randomised control trial of plasma exchange compared to standard of care in critically ill adults with severe COVID-19 associated respiratory failure, requiring supplemental oxygen or ventilatory support and elevated thrombo-inflammatory markers (LDH, CRP, ferritin, and D-Dimer). Patients randomised to receive PEX were treated with a daily single volume plasma exchange for a minimum of five days. Twenty-two patients were randomised of who 11 received PEX. Demographic and clinical characteristics were similar between groups at presentation. PEX was associated with a significant reduction in pro-thrombotic markers FVIII, VWF and VWF Ag: ADAMTS 13 ratio (*p* < 0.001). There were no differences in the reduction of inflammatory markers, severity of respiratory failure (*p* = 0.7), thrombotic events (*p* = 0.67), or mortality (*p* > 0.99) at 28 days. PEX successfully reduced pro-thrombotic markers, although was not associated with reduction in inflammatory markers, respiratory failure, or thrombotic events.

*Trial registration*: (NCT04623255); first posted on 10/11/2020.

## Introduction

The clinical syndrome associated with Coronavirus Disease 2019 (Covid-19) infection ranges from asymptomatic illness to acute respiratory distress syndrome, multiorgan failure, and death. COVID-19 is a unique thrombo-inflammatory disease, associated with an increased incidence of thromboembolic complications compared to patients with non-COVID-19 Acute respiratory distress syndrome (ARDS)^1,2^.

Patients with COVID-19 have elevated procoagulant factors including Von Willebrand Factor (VWF) and factor VIII, secondary to direct endothelial activation^3,4^, with higher levels associated with increased severity of disease^5^. Additionally, elevated proinflammatory cytokines are associated with increased mortality in COVID-19^6^.

The rationale for plasma exchange (PEX) in severe COVID-19 is to reduce both the thrombotic and inflammatory components associated with acute severe COVID-19, whilst not being directly immunosuppressive.

Our pilot case–control study suggested improved oxygenation, decreased incidence of acute kidney injury (AKI), improved lymphocyte counts and reduced circulating thrombo-inflammatory markers associated with PEX in patients with severe COVID-19^4^. We therefore conducted a prospective phase II randomised controlled trial in severe acute COVID-19 patients to investigate the efficacy and safety of PEX in addition to standard of care (SoC) in improving the thrombo-inflammatory effects of COVID-19.

Our primary objective was to assess the change in inflammatory and thrombotic markers associated with PEX. The secondary objectives were to assess the effects of PEX on progression of respiratory failure, cardiac dysfunction, acute kidney injury (AKI), incidence of acute thrombotic events, and 28- day mortality.

## Methods

### Study design

The COVIPLEX trial was phase II non-blinded randomised control trial of PEX and SoC compared to best SoC alone at a single site. Patients were randomised in a 1:1 ratio between October 2020 and October 2021. The trial underwent national ethical review (MREC20-EE-0164), and registered (with publication of the trial protocol) on Clinicaltrials.gov (NCT04623255); registered on 10/11/2020. Patients or their next of kin (where patients lacked capacity) provided written informed consent for the study. All methods were carried out in accordance with relevant guidelines and regulations. All experimental protocols were approved by national ethical review (MREC20-EE-0164).

### Patients

Participants screened included adult patients (> 18 years old) with polymerase chain reaction (PCR)—proven symptomatic COVID-19 infection. All patients were managed in a high dependency/intensive care setting requiring oxygenation (> 2L/min) to maintain oxygen saturations of ≥ 96%. Additionally, patients required at least two of the following raised markers; LDH > 2 times upper limit of normal, D-dimer > 2 times upper limit of normal, or CRP > 2 times upper limit of normal. Our feasibility study data and early papers from China related to the COVID-19 outbreak demonstrated that these parameters were associated with illness severity^4,7^. Cut- off values, required for defining inclusion criteria into the study, were based on levels on our pilot study.

Patients who were pregnant, or with significant past medical history e.g., patients with cancer or those not appropriate for invasive mechanical ventilation (because of frailty or burden of co-morbid disease as adjudged by treating clinicians) were excluded. Other exclusion criteria included patients with active bleeding, severe hypoxaemia (PaO_2_: FiO_2_ ratio < 100mmHg), significant cardiovascular support (noradrenaline requirement > 0.5mcg/kg/min), and allergy to Octaplas or excipients.

There were no criteria for the number of days from admission to PEX. However, daily identification of all new cases admitted to critical care or high dependency units, were approached. At least 24 h was given once patients received the study information.

### Randomisation

Randomisation and data collection were captured on Sealed Envelope (a randomisation and online data base for clinical trials). Randomisation was based on 20 patients with potential to increase to a total of 40 cases, dependent on safety committee review. Random allocation sequence was generated by the clinical trials team, and clinical investigators (who implemented the intervention) did not have access to the sequence until patients were assigned to the control or intervention groups. It was not possible for the clinical team to be blinded to the intervention.

### Standard of care

All patients received best SOC within a HDU/ICU setting. In contrast to the feasibility study when no routine additional treatments above SOC were given to patients^6^, and as a result of COVID 19 therapeutic guidance^8^ all patients in both arms received dexamethasone (8 mg daily for 10 days) and remdesivir^9^. From February 2021 tocilizumab was also introduced (covid19treatmentguidelines.nih.gov).

### Intervention

PEX was undertaken daily for 5 days, and could be repeated for a further 2 cycles at clinician discretion on agreement by at least 2 investigators. The exchange fluid was Octaplas LG plasma, single volume, 3 L per day for patients ≤ 90kg and 4 L per day if > 90kg. A central venous catheter was inserted to facilitate PEX.

### Data collection

Data on hospitalised patients were recorded from electronic healthcare records. Demographics, vaccination status, virus genotype, time of symptom onset, and COVID-19 treatments received were collected. Routinely collected biochemistry (including CRP, LDH, D dimer, ferritin), full blood count, coagulation markers (fibrinogen), cardiac biomarkers (Troponin, BNP), VWD screen (VWF antigen, VFW Activity, and Factor VIII), and ADAMTS 13 activity were recorded daily. Samples were collected pre- and post-PEX for measurement of VWD/ADAMTS 13 activity. Serum was collected for cytokine measurement at baseline and following completion of PEX treatment (or equivalent time point for the SOC group).

Adverse events related to vascular access insertion, plasma and exchange, thrombosis rates, respiratory support (e.g., need for mechanical ventilation), and mortality were documented. Patients were followed up to day 28 or death.

### Lab measurements

Routinely collected biochemistry, full blood count, coagulation, and cardiac biomarkers markers were measured by automated laboratory analysers. For patients undergoing PEX, coagulation markers (fibrinogen) and VWD screen (VWF antigen, VFW Activity, and Factor VIII), and ADAMTS 13 were recorded immediately before and after PEX, as well as the morning following the last PEX session. Biochemistry (including CRP, LDH, D dimer, ferritin), and full blood count were collected daily in the morning (prior to PEX sessions). For patients in the SoC group, all blood tests were evaluated from samples collected daily in the mornings. A blood sample for cytokine measurement was taken at baseline and on the morning after the last PEX session.

A four-plex panel (MesoScale Discovery, Rockville, MD, USA) was used to analyse IL-1b, IL-6, IL-10, and TNF-a as per manufacturer instructions. Ranges for serum cytokines among uninfected and asymptomatic individuals were determined from healthy healthcare worker volunteers in a previous study from our group^6^. VWF: antigen (Ag), activity, Factor VIII and ADAMTS 13 activity were collected pre and post PEX or daily in the SOC arm. All samples were then repeated days 14, 21 and 28.

### Outcomes

The primary outcome was the reduction in inflammatory markers associated with PEX, using a comparison of the binary outcome of 50% reduction in at least two inflammation markers (LDH, D-dimer and CRP) between the PEX + SOC and SOC trial arms. We assessed changes in biochemistry between enrolment and 24 h following the last PEX cycle that was administered (or equivalent timepoint for patients randomised to SoC).

The secondary objectives were to assess the effects of PEX on progression of respiratory failure, cardiac dysfunction, acute kidney injury, incidence of acute thrombotic events, and 28- day mortality. Respiratory support was scored on an ordinal scale from one to four (face mask oxygen = 1, high flow oxygen therapy or non-invasive positive pressure ventilation = 2, invasive mechanical ventilation = 3; extracorporeal membrane oxygenation = 4). Thrombotic events included the incidence of venous (deep vein thrombosis DVT or pulmonary embolism PE) or arterial thrombus (cardiac, neurological and peripheral vascular). Cardiac function was defined by cardiac biomarker measurements (Troponin-T and BNP). The incidence of acute kidney injury was defined by KDIGO serum creatinine criteria^10^. Analyses were conducted to evaluate the change in VWD markers and VWF ag/ADAMTS 13 activity ratio associated with PEX treatment.

### Statistics

Anonymized data were used for analysis. Continuous and categorical variables are reported as median (interquartile range) and n (%), respectively. Categorical data were compared using the chi-square test. Comparison of continuous and ordinal data between PEX and SoC groups was performed using the Mann Whitney U tests. Mann Whitney U tests was used to assess changes in coagulation markers (VWF antigen, VWF activity, ADAMST13 activity, and Factor VIII) before and after PEX sessions, and between the last PEX session and following 48 h.

A statistically significant difference between the treatment groups at the 5% level, assuming that PEX + SOC reduced inflammation markers in 80% of patients compared to just 20% for SOC if 20 patients were recruited (10 in each arm). The primary analysis involved a comparison of the binary outcome of 50% reduction in at least two inflammatory markers between the PEX + SOC and SOC trial arms using a chi-squared test. The risk difference (and ratio) was estimated with a 95% confidence interval. All analyses were performed on an intention to treat basis. Graphs were constructed, and statistical analysis performed using Stata and GraphPad Prism.

#### Role of funding source

The funding for the study was provided by Lifearc. The funder of the study had no role in study design, data collection, data analysis, data interpretation, or writing of the report. All authors had full access to all the data in the study and had final responsibility for the decision to submit for publication.

#### Ethical approval

All methods were carried out in accordance with relevant guidelines and regulations. All experimental protocols were approved by national ethical review (MREC20-EE-0164). Patients or their next of kin (where patients lacked capacity) provided written informed consent for the study.

## Results

### Baseline demographics, biochemistry, and treatment

**Figure 1:**
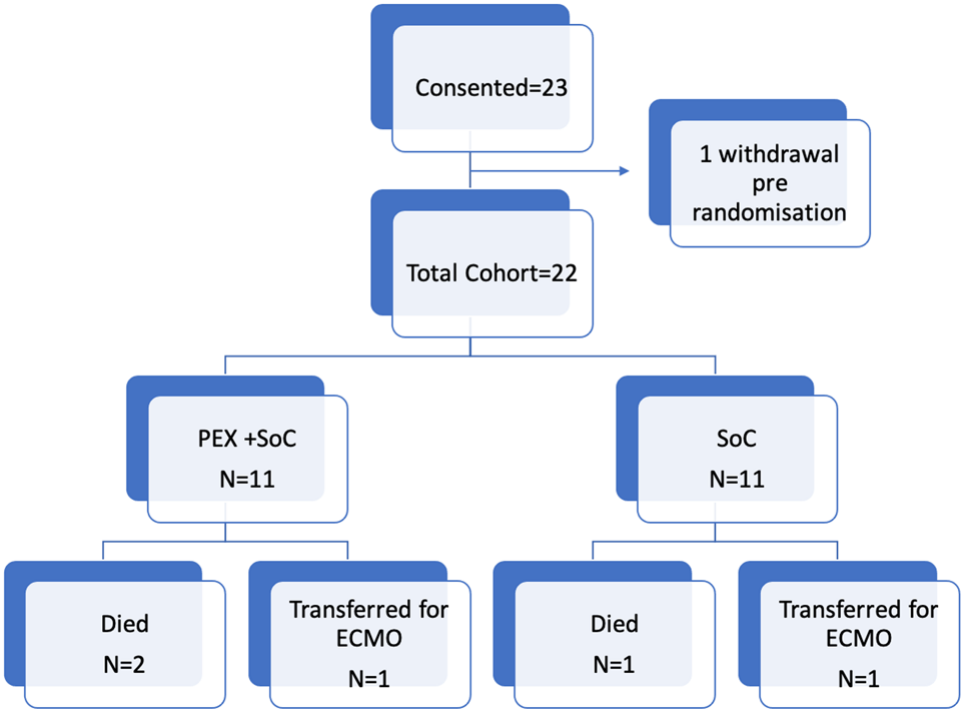
Consort diagram for enrolment and randomisation.

**Figure 2:**
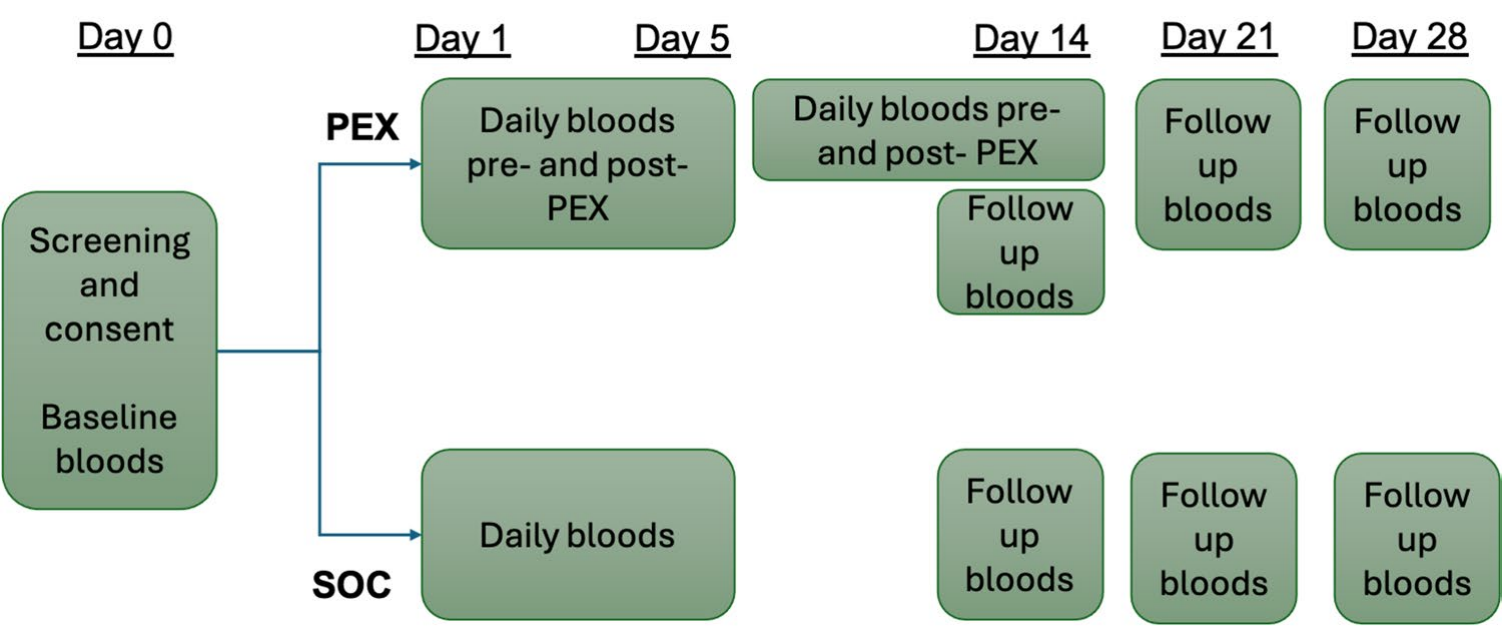
Patients in the PEX group had at least 5 days of PEX treatment. Six patients had additional treatment up to day 14. Blood samples were obtained daily on the first five days, on days 14, 21, and 28. Patients undergoing PEX had blood samples for assessment of coagulation markers taken before and after each PEX session. Otherwise, bloods were obtained in the morning alongside routine clinical bloods.

Recruitment was from October 2020 to October 2021. Twenty-three patients were consented to the study, one patient withdrawing before receiving treatment (Fig. 1). The median time from admission to consent was 1.5 days (range 0–8). Patients 1–6 had the initial SARS-COV-2 infection, patients 7–19 the alpha variant (B 1.1.7) and patients 20–23 the delta variant (B 1.617.2). The limited recruitment of patients was due to the decline in admissions with severe COVID-19 infection following the introduction of COVID 19 vaccination from December 2020 in the UK. A total of eleven patients were randomised to receive SoC and PEX each. The design/timing of PEX, blood sampling and clinical data collection is illustrated in Fig. 2. Baseline demographics and treatment are presented in Table 1**.**

**Table 1:** Baseline demographics, ventilatory status and routine laboratory parameters.

	SoC (n = 11)	PLEX (n = 11)
Age (years)	55 (42–69)	60 (56–65)
Gender (%male)	9 (82%)	9 (82%)
Ethnicity		
White	7 (64%)	4 (36%)
Asian	1 (9%)	4 (36%)
Black	2 (18%)	2 (18%)
Other	1 (9%)	1 (9%)
Body mass index (kg m^−2^)	29 (25–32)	30 (28–33)
Smoker		
Never	6 (55%)	5 (45.5%)
Ex	4 (36%)	5 (45.5%)
Current	1 (9%)	1 (0%)
Unknown	0 (0%)	1 (9%)
Co-morbid illness		
T2DM	3 (27%)	6 (45.5%)
HTN	2 (18%)	7 (64%)
IHD	1 (9%)	2 (18%)
Days symptoms to hospital admit	6 (4–10)	9 (7–10)
Vaccinated	1 (9%)	0 (0%)
COVID-19 variant		
Wild type	3 (27%)	3 (27%)
Alpha (B.1.1.7)	7 (64%)	7 (55%)
Delta (B.1.617.2)	1 (9%)	2 (18%)
Steroids	11 (100%)	11 (100%)
IL-6 inhibitor	4 (36%)	4 (36%)
Days Admit to PEX (or enrolment for SoC)	1 (1–4)	2 (1–3)
Days IL-6 inhibitor to PLEX (or enrolment for SoC)	0.5 (0–2)	1.5 (1–4.25)
Mode of ventilation Day 0		
Face mask	3 (27%)	2 (18%)
HFNO	1 (9%)	3 (27%)
NIPPV	6 (55%)	6 (55%)
IMV	1 (9%)	0 (0%)

Demographics, treatment, and level of respiratory support in patients randomised to standard of care or plasma exchange. (T2DM: type 2 diabetes mellitus, HTN: hypertension, IHD: ischaemic heart disease, IL-6: interleukin- 6, PEX: plasma exchange, SoC: standard of care, HFNO: high flow nasal oxygen, NIPPV: non-invasive positive pressure ventilation, IMV: invasive mechanical ventilation). Continuous and categorical variables are reported as median (interquartile range) and n (%), respectively. Categorical data were compared using the chi-square test. Comparison of continuous data between PEX and SoC groups was performed using the Mann Whitney U tests.

There were no differences in age, gender, BMI, smoking status, incidence of diabetes mellitus or ischaemic heart disease between patients enrolled to SoC and PEX. There were disproportionately more White patients (64% vs. 36%; *p* < 0.001) and fewer patients with hypertension (18% vs. 64% *p* = 0.030) among patients receiving SoC compared to PEX **(**Table 1**).**

The time from symptom onset to hospital admission was similar between patients in the SoC and PEX groups (6 (4–10) vs. 9 (7–10) days; *p* = 0.234). All patients in the SoC and PEX groups received steroids (100% vs. 100%; *p* = 1.000), and identical proportions received IL-6 inhibitors (36% vs. 36%; *p* = 1.000) with similar time between admission to receiving IL-6 inhibitors (0.5 (0.0–1.0) vs. 0.0 (0.0–0.75) days; *p* > 0.999) and between IL-6 inhibitor to enrolment to plasma exchange or SoC (0.5 (0.0–2.0) vs. 1.5 (1.0–4.3) days; *p* = 0.314). All patients received intermediate dose thromboprophylaxis, adjusted for renal function as per local Trust guidelines.

The mode of respiratory support on enrolment was similar between patients in the SoC and PEX groups (*p* = 0.532) with 6 of 11 (55%) patients in each group receiving NIPPV (non- invasive positive pressure ventilation). Serum creatinine at baseline was similar between patients receiving PEX and SoC. 71 (52–83) vs. 77 (46–85) umol/L; *p* = 0.793). A similar number of patients in the PEX (Stage 2 AKI = 1) and SoC (Stage 1 AKI = 1, Stage 3 AKI = 1) groups had AKI at baseline.

Coagulation, biochemistry, and haematology parameters on study enrolment between patients in the SoC and PEX groups (Supplementary Table S1) were comparable.

### Effect of PEX on coagulation markers

**Figure 3:**
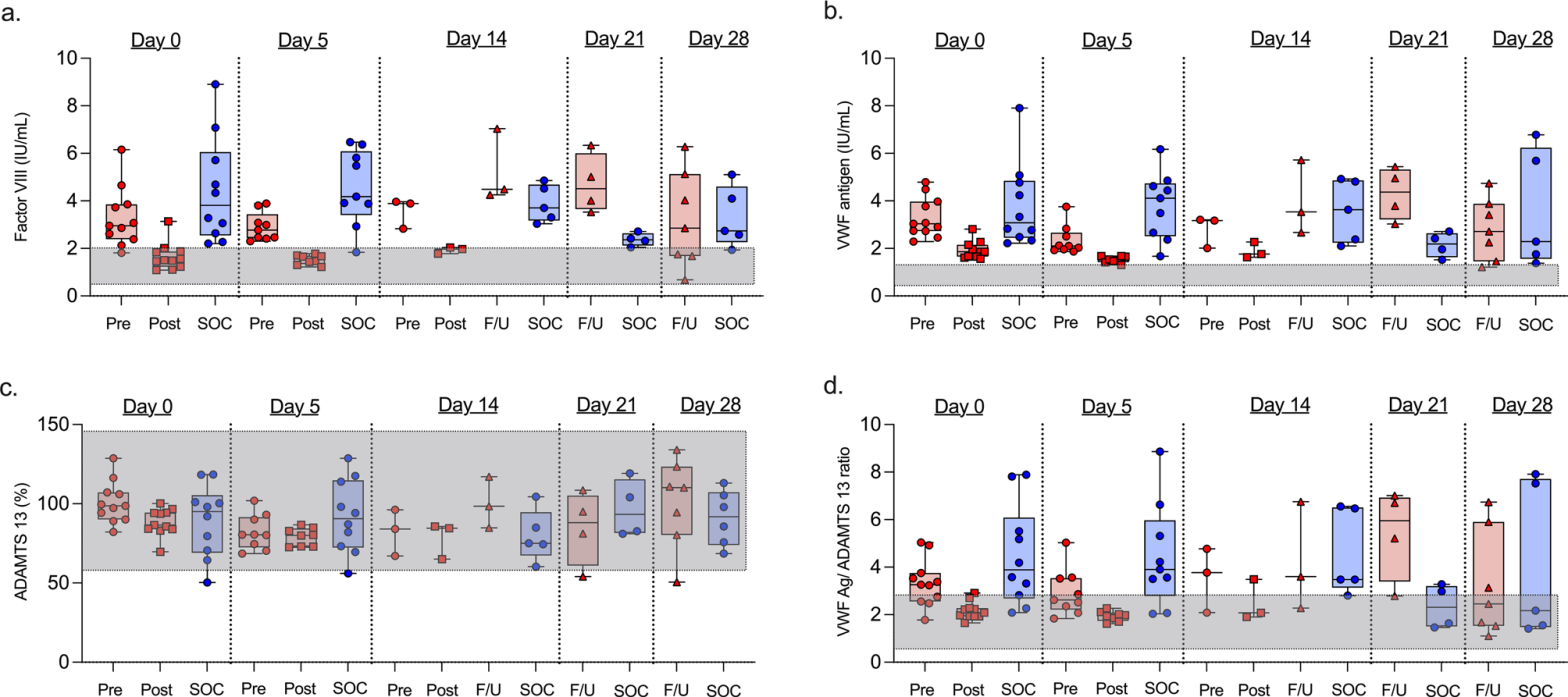
Levels of Factor VIII (**a**), VWF antigen (**b**) ADAMTS-13 activity (**c**), and VWF antigen: ADAMTS-13 ratio (**b**) in patients undergoing plasma exchange (PEX) and patients receiving standard of care (SoC) only. All ‘pre-PEX’ levels were taken immediately before PEX, and post- PEX levels were taken immediately after PEX. If patients were no longer undergoing PEX, their data are provided as ‘follow up’ (F/U) values, taken at pre-specified time points. Patients in the control group had blood samples taken at the same time points and values represented in the same graph. Results displayed as box and whisker plots with median values, interquartile range, and 95% confidence intervals for each time point, with individual patient data represented as a datapoint.

Samples were taken pre and post PEX while receiving treatment or daily for SOC. VWF antigen levels were significantly reduced from pre PEX day 1 to post PEX day 5 (3.04 to 1.57, *P* < 0.0001) and were lower than SOC (3.04 Vs 2.91 (*p* = ns) day 1 to 2.22 vs 3.68 day 5 (*p* < 0.0001). This effect was evident until day 14 (2.33 vs 3.22 *p* = 0.0066) between PEX and SOC, but thereafter there was no difference between either arm (Fig. 3). Comparable findings were seen for Factor VIII levels. VWF:Ag: ADAMTS13 activity ratio was significantly reduced with PEX compared to SOC and this effect was evident up to day 14. From pre PEX day 1 to post PEX day 5, the ratio reduced from 3.26 to 1.99 (*p* < 0.0001). There was a non- significant difference in the ratio between PEX and SOC on day 1 (3.26 vs 4.21 *p* = 0.133). However, by day 5, the ratio was significantly lower in PEX V s SOC (2.60 vs. 3.84 *p* < 0.0001). While the level remained lower in the PEX arm on day 14, the difference with SOC was not significant (Fig. 3).

### Effect of PEX compared to SoC on inflammatory parameters

The number of patients (excluding patients transferred for ECMO) who had a reduction of at least 50% for each of the individual marker was similar for both groups. However, when combined, 8/10 PLEX patients had two or more markers that achieved this, compared to 5/10 SOC patients. This was not statistically significant at the 5% level, mainly due to the small sample size. When ferritin was included in the analysis, 9/10 PLEX patients had two or more markers that achieve a 50% reduction, compared to 7/10 SOC patients, was although again not statistically significant, due to small numbers (Table 2).

**Table 2:** Inflammatory markers in PEX compared to SOC group.

Reduction of at least 50%	SoC (n = 10)	PLEX (n = 10)	*P*-value (χ^2^ test)
CRP	8 (80%)	7 (70%)	0.606
LDH	5 (50%)	6 (60%)	0.653
D-Dimer	4 (40%)	6 (66.7%)	0.245
At least two markers reduced by 50% (CRP, LDH, D-Dimer)	5 (50%)	8 (80%)	0.160
Ferritin	8 (80%)	9 (90%)	0.531
At least two markers reduced by 50% (CRP, LDH, D-Dimer and Ferritin)	7 (70%)	9 (90%)	0.264

A reduction of at least 50% of inflammation, comparing PEX to SOC. Although not statistically significant for any parameter, because of the small sample size, more cases in the PEX arm had a reduction in inflammatory parameters.

When analysed using mixed models, there was a significant reduction in LDH and ferritin for the PEX arm of 17.6% (95% CI 0.9–31.6%; *p* = 0.040) and 50.5% (95% CI 21.6–68.8%; *p* = 0.003) respectively. There was no significant difference for either CRP (*p* = 0.538) or D-Dimer (*p* = 0.930).

**Figure 4:**
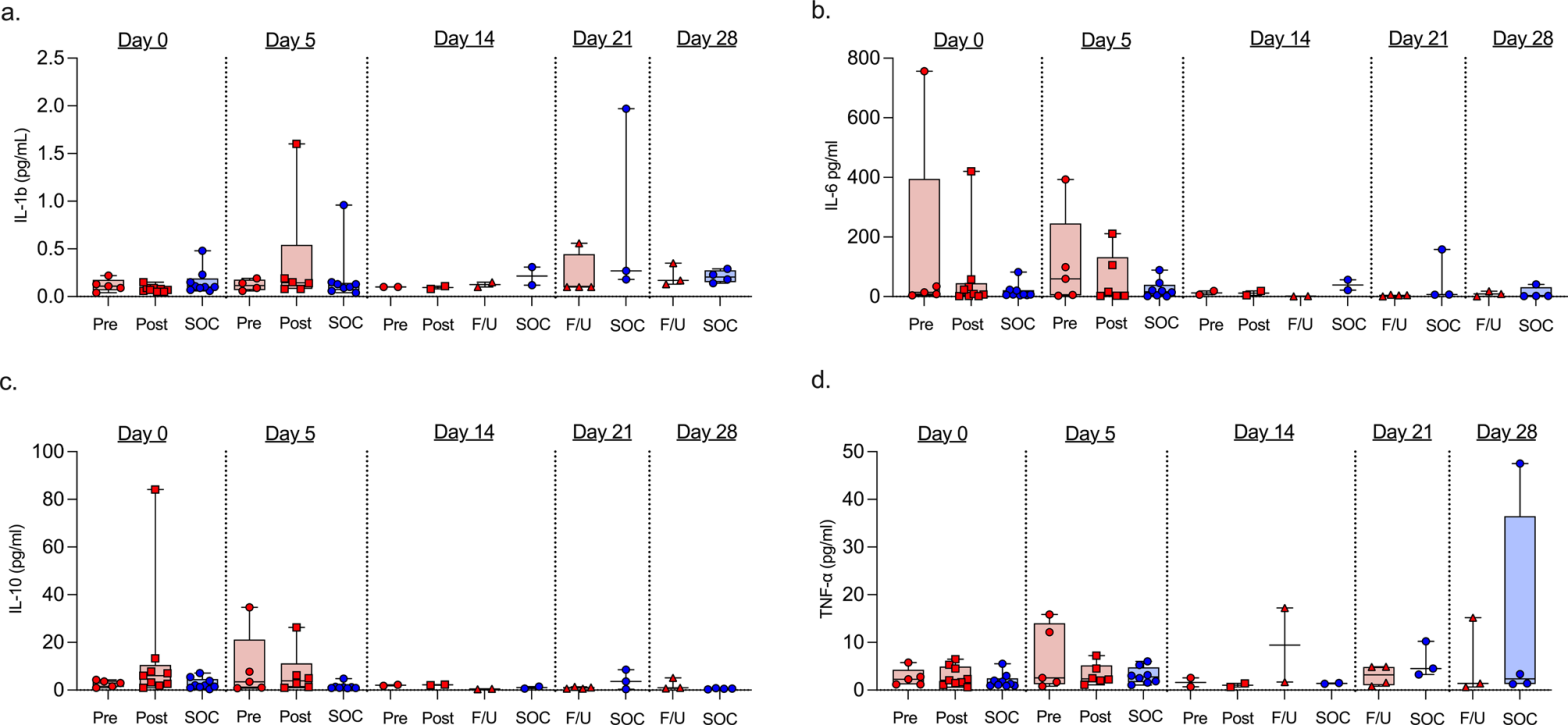
Levels of cytokines IL-1beta (**a**), IL-6 (**b**), IL-10 (**c**), and TNF-alpha (**d**) in patients undergoing plasma exchange (PEX) and patients receiving standard of care (SoC) only. Note different Y axis scales. Results displayed as box and whisker plots with median values, interquartile range, and 95% confidence intervals for each time point, with individual patient data represented as a datapoint.

Four cytokines were analysed: IL-10, IL-1B, IL-6 and TNF-alpha (Fig. 4). There were no differences in serum cytokines before and after PEX.

### Clinical outcomes at day 28

One patient in the SOC group and 2 patients in the PEX group died within 28 days. One patient in each group were transferred to ECMO within 28- days. Of the remaining patients, one of 11 patients receiving PEX and 4 of 11 patients in the SOC group were lost to follow up a on day 28.

There were 8 thrombotic episodes documented in the cohort. Within the SOC arm, 2 PEs were at presentation of COVID19 and before randomisation; 1 popliteal artery occlusion was confirmed 2 days following randomisation. In the PEX arm, 2 PEs were present at randomisation and 1 above knee thrombosis (1 week following randomisation) and 2 cases of PE (7- and 19-days post randomisation) were documented in association with severe COVID-19 lung changes. There was no difference in the incidence of thrombotic episodes at day 28 between patients receiving PEX or SoC (Table 3).

**Table 3:** Secondary outcomes.

	SoC (n = 11)	PLEX (n = 11)	*p*-value
Serious adverse events	1	0	> 0.999
Requirement for IMV			
28 days	4 (36%)	6 (55%)	0.670
Time to IMV (days)	3 (-4 to 6)	5 (1–9)	0.224
Requirement for ECMO			
28 days	1 (9%)	1 (9%)	> 0.999
Requirement for RRT			
28 days	1 (9%)	3 (27%)	0.587
Time to RRT (days)	18	12 (8–14)	–
Mortality			
28 days	1/11 (9%)	2/11 (18%)	> 0.999

*IMV* invasive mechanical ventilation, *ECMO* extracorporeal membrane oxygenation, *RRT* renal replacement therapy, *PEX* plasma exchange, *SoC* standard of care.

There was no difference in the level of respiratory support at day 28 between patients receiving PEX or SoC (3 (2–3) vs. 2 (2–3) *p* = 0.717)). Requirement for invasive mechanical ventilation by day 28 was not statistically significant between patients receiving PEX or SoC (55% vs. 36%; *p* = 0.670). The time to requiring IMV was not statistically significant between groups (5(1–9) days vs. 3 (−4 to 6) days; *p* = 0.224). One patient in the SoC group and PEX group were transferred for ECMO for severe respiratory failure on days 2 and 16 respectively (Table 3).

Baseline creatinine was similar between SOC and PEX groups as above. Two patients randomised to SoC and 1 patient randomised to had stage 1 AKI on enrolment (*p* = 0.534). By day 28, 3 of 10 patients in the SoC group developed AKI (Stage 1 AKI = 1, Stage 3 AKI = 2) and 1 of 11 patient in the PEX group developed AKI (Stage 3) (*p* = 0.223) Among patients who developed AKI, peak creatinine was mostly beyond day 14 (Table 3). Cardiac biomarkers were similar between groups at baseline and on follow up.

Regarding safety, there was only one SAE in relation to hepatitis in a patient who received tocilizumab in the SOC arm. All remaining AEs and SAEs were expected from severe COIVD 19 infection. There were no adverse events in relation to plasma exchange or the use of Octaplas.

## Discussion

Acute, severe COVID-19 is associated with a thrombo-inflammatory pathology and high morbidity and mortality rates. Within the initial wave, prompt investigative studies were undertaken. The 3 main additional treatments used in conjunction with SOC within this cohort but not in our pilot study included dexamethasone, remdesivir and, latterly, tocilizumab. Dexamethasone was associated with a reduced mortality in those receiving oxygen or requiring invasive ventilation^8^, remdesivir reducing time to recovery in hospitalised patients^11^, while tocilizumab reduced progression to the mechanical ventilation and improved survival^12^.

We demonstrate that levels of factor VIII and VWF antigen are elevated in acute COVID-19, and can be safely and effectively be reduced by PEX. More patient on PEX + SOC had > 50% reduction in at least two inflammation markers than those on SOC alone, but this was not statistically significant, because of the patient numbers.

Levels of inflammatory markers including elevated LDH, ferritin, CRP, IL-6, D-dimer, and lower levels of platelets and lymphocytes are associated with mortality among patients with COVID-19^13^. This may represent an exaggerated host immune response to the viral infection. The reduction in D-dimer, IL-6, LDH and ferritin and increase in lymphocytes and ADAMTS-13 activity associated with PEX treatment has been described in another RCT^14^.

Trials of multiple therapeutic approaches have been undertaken, primarily focusing on immunosuppressive therapies^15^. Our rationale for plasma exchange in severe COVID-19 is to reduce both the inflammatory and thrombotic components associated with acute COVID-19 whilst not being directly immunosuppressive. The potential benefit of plasma exchange (PEX) in severe COVID -19 infection has previously been described in cases, cohorts and small studies and its overall utility presented in systematic review between March, 2020 and May, 2021^16^.

COVID-associated coagulopathy has been described as a novel syndrome^17^. Despite anticoagulation, a high number of patients with ARDS secondary to COVID-19 developed life-threatening thrombotic complications^2^. The widespread pulmonary macro- and microthrombi noted at post-mortem implicates the prothrombotic component as a major factor underlying the pathophysiology of this disease^18,19^. Prothrombotic factors, including factor VIII, and VWF antigen are elevated in patients with COVID-19^4,20^. Higher VWF/ADAMTS 13 ratios have been associated with very severe COVID-19^3^. We demonstrated a reduction in VWF antigen and factor VIII levels with plasma exchange and a reduction in the VWF Ag: ADAMTS13 ratio with PEX. PEX reduced levels from that noted in moderate to severe disease and therefore worse outcomes to median levels relating to less severe outcomes. These differences in VWF Ag: ADAMTS 13 ratios and mortality and morbidity have been previously documented^3^.

The potential benefits of PEX in COVID-19 may be offset by the proposed removal of COVID-19 antibodies. Others have described the detection of SARS-CoV-2-specific IgG and IgA antibodies in the waste bag plasma and a reduction the circulating amount of antibodies by one log order^21^. Additionally, the effect of PEX on removal of therapeutic agents, particularly monoclonal antibodies including Tocilizumab, cannot be overlooked. We delayed PEX for 24 h following administration of an IL-6 receptor antibody.

This study was an open-label, single-centre study, and the study achieved the minimum required numbers based on declining SARS-CoV- 2 admissions. However, the randomisation achieved a well-matched population both demographically and for severity of disease.

Greater severity of COVID-19 is associated with higher levels of thromboinflammatory markers (VWF ag/ADAMTS 13 activity level), the latter which can be reduced by PEX. To determine if the thromboinflammatory markers play pathogenic role, we conducted a clinical trial to assess if reducing these mediators using PEX was associated with in improvement in disease severity. However, due to the relatively small sample size, we were unable to ascertain any potential clinical effect of reducing thromboinflammatory markers.

All patients received empirical and supportive ICU therapy including intermediate dose anticoagulation with enoxaparin and dexamethasone. Four of eleven patients in each group received IL-6 inhibitors (e.g., Tocilizumab). This was in contrast to our pilot study, in which patients did not receive steroids or IL-6 inhibitors. Levels of IL-6 above 3.27 pg/ml using this cytokine assay were noted to be associated with severe COVID-19 disease and more likely to require intubation and ventilation^22^. The change in the standard of care for COVID-19 reflects the rapidly changing evidence in the management of COVID-19, and may explain why the results in this RCT did not reflect changes seen in our earlier pilot study. Additionally, there were differences in virus genotype throughout the study, which is known to have an influence on illness severity and outcome^23^.

In conclusion this phase II randomised controlled study of PEX compared to SOC demonstrated the safety of PEX in acutely unwell patients with severe COVID-19. We show reduction of elevated Factor VIII and VWF and VWF Ag:ADAMTS 13 ratio in the context of persistent inflammation, and the potential for PEX to safely and effectively reduce pro-thrombotic mediators in COVID-19.

## Data availability

Anonymised data can be available under reasonable request. The corresponding author should be contacted.

### Supplementary Information

Supplementary Information.
