# Supplementary material for: A randomised controlled trial of plasma exchange compared to standard of care in the treatment of severe COVID-19 infection (COVIPLEX)
Source: Sci Rep. 2024 Jul 23;14:16876. doi: 10.1038/s41598-024-67028-3 (PMC11266620; doi:10.1038/s41598-024-67028-3)
Supplement: Supplementary file 1 — Supplementary Information. [file 41598_2024_67028_MOESM1_ESM.docx]

**Supplementary Material**

| Screening blood results | SoC | | | PLEX | | |
| --- | --- | --- | --- | --- | --- | --- |
|  | N |  | Median (IQR) | N |  | Median (IQR) |
| WCC (x 10^9^/l) | 11 |  | 9.97 (7.51) | 11 |  | 6.18 (2.4) |
| RCC (x10^12^/l) | 11 |  | 4.43 (0.73) | 11 |  | 4.62 (1.35) |
| Haemoglobin (g/l) | 11 |  | 125 (24) | 11 |  | 130 (33) |
| HCT (l/l) | 11 |  | 0.371 (0.073) | 11 |  | 0.382 (0.1) |
| MCH (pg) | 11 |  | 29.7 (3) | 11 |  | 29.4 (4.8) |
| MCV (f l) | 11 |  | 86 (8) | 11 |  | 85 (12.6) |
| Platelets (x10^9^/l) | 11 |  | 317 (131) | 11 |  | 228 (124) |
| Neutrophils (x10^9^/l) | 11 |  | 8.92 (7.25) | 11 |  | 5.41 (2.42) |
| Lymphocytes (x10^9^/l) | 11 |  | 0.6 (0.18) | 11 |  | 0.69 (0.46) |
| Monocyte (x10^9^/l) | 11 |  | 0.29 (0.25) | 11 |  | 0.23 (0.31) |
| Eosinophils (x10^9^/l) | 11 |  | 0.01 (0.07) | 11 |  | 0 (0.01) |
| Basophils (x10^9^/l) | 11 |  | 0.01 (0.01) | 11 |  | 0.01 (0) |
| PT (s) | 11 |  | 11.4 (0.9) | 10 |  | 11.05 (0.6) |
| APTT (s) | 11 |  | 30 (10) | 10 |  | 31.5 (4) |
| Fibrinogen (g/l) | 8 |  | 6.37 (1.955) | 10 |  | 5.355 (2.5) |
| ALT (iu/l) | 11 |  | 33 (14) | 11 |  | 42 (27) |
| ALP (iu/l) | 11 |  | 71 (67) | 11 |  | 74 (40) |
| Bilirubin (umol/l) | 11 |  | 10 (8) | 11 |  | 8 (4) |
| Sodium (mmol/l) | 11 |  | 137 (5) | 11 |  | 135 (4) |
| Potassium (mmol/l) | 11 |  | 4.5 (0.6) | 10 |  | 4.6 (0.6) |
| Urea (mmol/l) | 11 |  | 6.4 (9.3) | 9 |  | 6.4 (3.7) |
| Creatinine (umol/l) | 11 |  | 77 (51) | 11 |  | 70 (24) |
| EGFR | 11 |  | 90 (9) | 11 |  | 90 (0) |
|  |  |  |  |  |  |  |
| Inflammatory markers |  |  |  |  |  |  |
| CRP (mg/l) | 11 |  | 112.7 (132.4) | 11 |  | 167.9 (79.9) |
| LDH (iu/l) | 11 |  | 528 (178) | 11 |  | 601 (385) |
| D-Dimer (ug/l) | 11 |  | 2420 (8330) | 11 |  | 1660 (19660) |
| Ferritin (G/L) | 10 |  | 1113 (520) | 11 |  | 1204 (1350) |
|  |  |  |  |  |  |  |
| Secondary outcomes |  |  |  |  |  |  |
| BNP (ng/l) | 10 |  | 229 (385) | 9 |  | 167 (710) |
| Troponin (ng/l) | 11 |  | 9 (19) | 10 |  | 7.5 (4) |

**Supplementary Table 1**: Screening blood results at baseline. Values are numbers of patients and median (inter-quartile range).
